# Incidence of Morphological Defects in Sperm of Mice Exposed to Hospital Effluent

**DOI:** 10.3390/toxics11050418

**Published:** 2023-04-29

**Authors:** Priyanka Mathur, Kusum Rani, Pradeep Bhatnagar, Swaran Jeet Singh Flora

**Affiliations:** 1 Department of Environment and Life Sciences (Zoology), IIS (Deemed to be University), Jaipur 302020, India; goyalkusum228@gmail.com (K.R.); pradeep.bhatnagar@iisuniv.ac.in (P.B.); 2 Department of Pharmacy, Era Medical University, Lucknow 226002, India; sjsflora@hotmail.com

**Keywords:** hospital liquid effluent, reproductive toxicology, sperm deformities, sperm morphometrics

## Abstract

Hospital effluents are loaded with drugs, radioactive elements, pathogens, etc. Effluents from treatment plants at source sites may get mixed up with potable water, leading to numerous detrimental/toxic effects. In this study, efforts were made to investigate the toxic effects of one such effluent from a local hospital on the reproductive characteristics of mice when orally administered daily for 60 consecutive days. We primarily focused on the changes in the morphology of the sperm and its geometric morphometrics, i.e., sperm head length and width, area, and perimeter, measured using ImageJ software. The incidence of sperm defects was recorded, and variations in the morphometrics were analyzed by one-way ANOVA using Tukey’s post hoc test. A physico-chemical characterization of the water samples was also performed to assess the basic water quality. In summary, the study revealed the critical role of treated water in inducing different abnormalities in sperm, such as the absence of a head, bent necks, abnormal neck attachment, highly coiled tails, and missing tails. Significant differences (*p* < 0.01 **, *p* < 0.001 ***) in the morphometrics of spermatozoa with banana heads, hammer heads, missing heads, pin heads, and missing hooks were noted compared to corresponding controls. It could thus be concluded that treated hospital effluent is still inadequately clean and contains significant amounts of toxicants that might be detrimental to sperm quality.

## 1. Introduction

A typical sewage treatment plant in a hospital is designed to remove various toxicants from its liquid discharge but at times fails to eliminate micropollutants such as pharmaceutically active compounds, endocrine-disrupting compounds (EDCs), and hormones [1]. These pollutants might be hazardous when they percolate into watersheds or penetrate into natural habitats, leading to the contamination of ground water and drinking water. This may pose serious harm to human health and the aquatic ecosystem [2,3].With the objective of finding a plausible explanation for reproductive toxicity in mice, we tested hospital effluent that was supposedly treated. We determined the physico-chemical characteristics of water samples to assess the basic water quality and examined changes in sperm morphology and morphometry using mice as an experimental model. Alabi and Shokunbi [4] and Jirova et al. [5] have also reported on the estrogenic and androgenic properties of wastewater samples due to the presence of a heterogenous mixture of toxicants, which seems to be responsible for male infertility defects.

Sperm morphology is believed to be a strong indicator of an individual’s testicular health. It correlates with the physiological and environmental stress that affects the body’s physiology without influencing the overall health of an individual [6,7]. Some of the micropollutants present in hospital effluents have the potential to interfer with important physiological processes such as the morphological development of the urinogenital system, affecting secondary sexual traits, mimicking naturally occurring hormones such as androgens, impacting the binding of endocrine disruptors to the corresponding receptors, and inhibiting enzymes such as 5α-reductaseand aromatase [8]. Spermatogenesis, which is a multi-step process, leads to the generation of spermatozoa from spermatogonia via mitosis, meiosis, and spermiogenesis. During this complicated process, type-A spermatogonia located in the basal compartment of the seminiferous tubules undergo mitosis and differentiate to form type-B spermatogonia, which further differentiate into primary spermatocytes. Following meiosis, the primary spermatocytes develop into haploid spermatids, which undergo extensive morphological and molecular changes to develop into spermatozoa via spermiogenesis. During this whole process, any change in the hormonal level, damage to the integrity of the blood–testis barrier (BTB),or disruption in the process of the maturation of spermatids due to toxicants may lead to morphological defects in the sperm [9].Thus, we considered it useful to study the effects of the heterogenous mixture of toxicants present in effluents on the morphology of sperm by identifying defects in the shape of spermatozoa in mice using geometric morphometrics.

## 2. Materials and Methods

### 2.1. Sample Collection

The water samples were collected from a major hospital situated in the Sitapura area of Jaipur city, Rajasthan, India. Samples were collected from a discharge point of the hospital where a treatment plant was installed to remove pollutants. Samples were collected in pre-cleaned and sterilized glass bottles during the peak hours of hospital activity, i.e., between 11:00 A.M. and 1:00 P.M. Throughout the study, samples were kept at 4 °C and were used in their undiluted form. A physico-chemical characterization of the water samples was carried out to assess their quality, and the results are summarized in Table 1.Severalvariables for determining the water quality were considered based on the standard methods of the American Public Health Association(APHA) (2005) and Maiti (2004) [10,11].

### 2.2. Experimental Animals

Sexually developed Swiss albino male mice (*Mus musculus*) ranging in weight from 25 to 30 g were used for this study. The animals were acclimatized for a week prior to the start of the experiment in a well-ventilated pathogen-free animal house at the IIS (Deemed to be a University), Jaipur, Rajasthan, India. The animal facility of the department is registered with the Committee for the Purpose of Control and Supervision of Experiments on Animals (CPCSEA), Government of India (Registration No. 1689/PO/a/13/CPCSEA). The experimental protocol of the study was approved by the Institutional Animal Ethical Committee (Letter # IAEC/2019/II/1). The animals were fed with standard food pellets, and water was provided ad libitum.

The animals were divided into two groups of 10 animals each. The control group (Group I) was given potable water, and the treatment group (Group II) was administered treated/recovered hospital effluent in its unaltered form for 60 consecutive days through the oral route.

### 2.3. Sperm Morphology Test

The test was carried out as formerly described by Wyrobek et al. [12]. After the completion of the exposure period (60 days), the animals were euthanized by cervical dislocation, ensuring minimal trauma and pain for the animals, as suggested by the IAEC. Blood and tissues were collected for various investigations. In the present study, only cauda epididymes were used for conducting sperm morphology tests. The cauda epididymes were carved out and minced with a fine needle in 2ml of phosphate-buffered physiological saline. A fraction of suspension was then mixed with 1% Eosin Y (H_2_O), and after 30 min, smears were produced on grease-free clean slides and allowed to dry for 45 min. Slides were mounted on the mounting medium and observed for sperm abnormalities. A total of 1000 spermatozoa per animal were examined under the phase-contrast microscope (Nikon) fitted with a camera (model no. E201) at 400-fold magnification, and each sperm and its anomalies were identified following strictly defined criteria [13,14]. All the findings are presented in Table 2.

### 2.4. Geometric Morphometrics of Sperm

To identify shape differencesbetweenthe spermatozoa of the control group and the treated group, basic morphometric parameters such as sperm head length, head width, perimeter, and area were measured using standard formulae, as presented below:

- Head length (µm): maximum length of main axis of head from basal position to apical position.
- Head width (µm): maximum head width from dorsal to ventral side of the sperm.
- Sperm perimeter (µm): boundary that surrounds the sperm.
- Sperm area (µm^2^): area occupied by the sperm.

The measurements werecarried outusing ImageJ software for image analysis. These analyses were used to identify the changes in sperm shape that resulted from the action of the heterogenous mixture of toxicants present in the watersamples. Comparisonsbetween groups were carried out by Student’s t-test, and significant differences amongst the mean values of different groups were further analyzed by a one-way ANOVA (Tukey’s) test using GraphPad prism 8 software, as shown in Table 3. Statistical significance was adjusted to *p* < 0.05 *, *p* < 0.01 **, and *p* < 0.001 ***.

## 3. Results

### 3.1. Physico-Chemical Analyses

Prior to the assessment of the sperm defects, the samples were first tested regarding their physico-chemical characteristics, and the results are presented in Table 1.

The pH of the water samples was 8.4, which was within the acceptable limits (5.5–9.0). The temperature of the water samples ranged between 24 and 26 °C, which was also considered to be well within the permissible limits. However, much higher values of chlorides were noted in the treated effluents, indicating their abundant use in the purification process. The total suspended solids (TSS) and total dissolved solids (TDS) values of the treated effluents were found to be higher than the standard limits.

The values of dissolved oxygen (DO) and biological oxygen demand (BOD) in the water samples were within the permissible limits.

### 3.2. Sperm Morphology Analyses

After the physico-chemical characterization, the water samples were used for sperm morphology tests. Abnormal sperm morphologies were observed in the treated mice. These included head abnormalities such as banana heads; hammer heads; large heads; missing heads; hookless heads; and amorphous heads (knob heads, umbrella heads, pointed heads, and pin heads).They also included neck and mid-piece defects, such as bent necks, spermatozoa with kinks, and abnormal neck attachments, and tail defects, such as broken tails, distal bent tails, schizoid tails, tails with hairpin loops, highly coiled tail sperm without tails, and the presence of light and heavy cytoplasmic droplets in tails. These were the notable abnormalities observed in the spermatozoa of the treated-group animals. All defects observed in our study and detailed explanations of each defect are provided in Table 2.

### 3.3. Geometric Morphometrics of Sperm

A significant increase of 3.1 µm in the head length of banana-head sperm and significant decreases of 1 µm, 1.4 µm, and 1.6 µm in the head length of hammer-head, pin-head, and umbrella-head sperm, respectively, were observed at *** *p* < 0.001 when compared with the values of the spermatozoa from the control group. There was a significant increase of 2.6 µm in the head width of umbrella-head sperm when compared with the control group (*** *p* < 0.001). Significant decreases (*** *p* < 0.001) of 30.8 µm, 24.4µm, and 31.3 µm in the sperm perimeter values of the hammer-head, pin-head, and umbrella-head sperm, respectively, were observed when compared with the control values. Likewise, the area of the banana-head sperm was 13.6 µm lower than the control value, which represented a significant decrease at *p* < 0.01; the trend was similar in headless (**p < 0.01), pin-head, and hookless spermatozoa (*** *p* < 0.001).

## 4. Discussion

Hospital liquid effluents contain wide varieties of toxic substances that include chemicals, radioactive substances, pharmaceuticals, pathological wastes, and nano-sized metals. These effluents, in general, are discharged into municipal sewer pipelines, sometimes without treatment, resulting in the mixing of untreated or partially treated water with the municipal water supply, leading to the contamination and pollution of potable water [29]. In the present study, the presence of toxicants in the water samples and their adverse reproductive effects in a murine model were studied. The study specifically focused on the effects of toxicants as a whole on the morphology of the sperm, and we used geometric morphometrics to clearly identify shape differences in mice spermatozoa obtained from both control and treated groups. Due to a technical problem, we could not determine various heavy metals and other toxicants in the water samples, which might have been the causative factor for the morphological defects.

The morphology of sperm is an important variable often correlated with male reproductive disorders. It can influence fertility, because sperm must have a certain shape to be able to penetrate the outer layers of the ovum. Micropollutants such as pharmaceutically active compounds, endocrine-disrupting compounds (EDCs), and hormones sometimes remain in treated wastewater, despite the assumption that they have been removed by wastewater treatment processes. These can also interfere with developmental processes and deteriorate the homeostasis of the living body [30]. Some nano-sized metals also remain in wastewater after treatment, leading to the increased production of reactive oxygen species (ROS), which affect lipid peroxidation and result in an intracellular oxidative burden, particularly in membrane lipids. Membrane lipids are required to provide plasma with membrane fluidity, a crucial factor for sperm motility; structural integrity; and, eventually, sperm viability. Spermatozoa are highly susceptible to the deleterious effects of ROS due to the large amounts of unsaturated fatty acids found in their cell membranes [31,32]. Besides this, the nature of initial sperm–zona pellucida interactions relies primarily on the recognition of carbohydrate moieties present on the zona pellucida by lectin-like binding receptors on the sperm head (carbohydrate-dependent model). This process is highly sensitive and can be disrupted by toxicants [33], resulting in teratozoospermia.

There are reports demonstrating that teratozoospermia is not the only critical factor for success in fertilization and pregnancy outcomes. There are many other factors that can affect the fertilization process. Several other studies have suggested that sperm morphological defects may be associated with not only penetration but also embryonic development, because sperm presenting normal acrosome function may still contain ultra structure aberrations and dysfunction that will adversely affect embryo quality. In order to gain a better understanding of the influence of sperm morphology on fertilization, further research is urgently needed [34].

In this study, mouse sperm obtained from the cauda epididymes of the control group were found to be normal with standard sperm morphometric parameters, but in the experimental group, notable sperm morphological defects were observed. Head abnormalities such as banana heads, hammer heads, large heads, pointed heads, missing heads, pin heads, hookless heads, knob heads and umbrella heads were observed in the treated group. Defects in the sperm head can disturb the ability of the sperm to reach and penetrate the egg. Defects such as bent necks, spermatozoa with kinks, and abnormal neck attachments were seen after treatment with water samples. Such mid-piece defects in sperm might be associated with defective mitochondria; likewise, centrioles, which form the guidance system for moving chromosomes and are stored in the sperm neck, might be missing or broken if there is a defect in the mid-piece of a sperm. Tail defects such as broken tails, distal bent tails, schizoid tails, tails with hairpin loops, highly coiled tails/dag-defect sperm, and sperm without tails were also observed in the experimental animals. Such abnormal tail structures can be correlated to sperm motility disorders, thereby reducing the chance of natural conception. In many cases, spermatozoa with cytoplasmic droplets (CDs) were observed. These droplets represent a smear of cytoplasm that primarily remains attached to the neck region and, during the epididymal transit of the sperm, progressively shifts its position to the back end of the mid-piece. The droplet is shed when the sperm leaves the caput epididymis and arrives at the cauda epididymis. Spermatozoa that contain extra cytoplasm have repressed motility. In the present study, the increased retention of cytoplasmic droplets by cauda epipidymal sperm was observed. The pollutants present in wastewater might inhibit the shedding of cytoplasmic droplets. However, we are currently not in a position to highlight the exact mechanism behind this, which requires more extensive studies.

An increased number of abnormally shaped sperm actively affects the fertilization rate and further reduces the viability and motility of sperm. When the sperm cells were exposed to water samples, sperm with abnormal morphologies were noted, suggesting that these sperm may not reach the oviduct or participate in fertilization [4]. To increase the sensitivity of the tests, the geometric and morphometric assessment of the sperm helped in identifying the effect of reprotoxicants. This tool improved the analysis of the sperm head morphologies, which was based on the exact spatial position of a given anatomical structure [35]. Briefly, the overall impact of the water samples on the morphology of the sperm indicated a deterioration in sperm quality in terms of sperm morphological defects, suggesting shortcoming, lacunas, or defects in the water treatment process.

## 5. Conclusions

Based on the data collected from this study, it could be concluded that the incidence of sperm morphological defects may have been caused by the hospital effluent, which was purported to be safe by the hospital administration after undergoing a treatment process. This study also indicated that recovered hospital effluents still have properties that may cause reproductive toxicity. There is thus a need to ensure the efficacy of treatment plants at healthcare establishments and guarantee that the proper analysis of wastewater is carried out before its disposal.

## Figures and Tables

**Table 1:** Physico-chemical characterization of the water samples.

Parameter	Treated Hospital Effluent	General Standards for Discharge of Pollutants (APHA, 2005)	
		Inland Surface Water	Irrigation Water
pH	8.4	5.5–9.0	5.5–9.0
Temperature	24	^_^	-
Chlorides	15.32	1.0	-
TS	2.0	-	-
TSS	1864.2	100	200
TDS	2450	1500	-
DO	4	4	-
BOD	1.5	3	100

All the values are expressed in milligrams per liter, except for pH (unitless) and temperature (in Celsius). TS: total solids, TSS: total suspended solids, TDS: total dissolved solids, DO: dissolved oxygen, BOD: biochemical oxygen demand.

**Table 2:** Detailed explanation of head, mid-piece, and tail abnormalities observed in sperm of mice treated with hospital effluent.

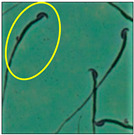 Normal spermatozoa	Normal sperm morphologies with a hook-shaped head, unbroken or complete mid-piece, and straightened or uncoiled single tail were observed in the mice of the control group. With this intact structure, the sperm were healthy and could swim well.
Various head defects were observed in the mice of the experimental group.	
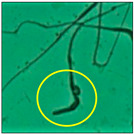 Banana-head	Banana heads, also known as tapered heads/thin narrow heads and look “cigar-shaped”, were observed. The tip of the head had a reduced hook. Such a defect can occur due to broken DNA, abnormal chromatin, the packaging of the paternal DNA genetic material, or an abnormal number of sperm chromosomes. With this defect, spermatozoa generally do not undergo acrosome reactions or may be detrimental to embryo development [15].
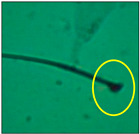 Hammer-head	Hammer-shaped sperm heads that looked hookless and small were observed in the animals of the experimental groups. Such a defect can occur when there is a premature breakdown in the nucleus or when the inner parts of the sperm head are missing [16]. This defect prevents acrosome formation, makes acrosomal reactions impossible or aberrant, hinders gamete interaction, and leads to an incomplete fertilization process.
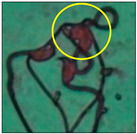 Large-head	Megalo-headed spermatozoa, i.e., presenting giant heads, were also observed in the experimental animals. Such sperm carry extra chromosomes [17], which cause anomalies and problems in fertilizing eggs. This defect can be genetic or can occur due to toxicants.
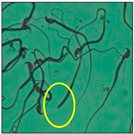 Headless	A large number of decapitated/acephalic spermatozoa were noted in this work. This defect presented as a tail without a head. According to Yan (2015) [18],the sperm neck becomes fragile or is lost, and this leads to the detachment of the head from the tail.
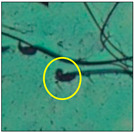 Hookless	In some spermatozoa, the absence of an apical hook and an incomplete acrosome were observed. Such a defect makes acrosomal reactions impossible or aberrant, which is responsible for hindering the fertilization process [19].
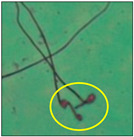 Pin-head	Pin-head sperm were also noticed during the study. This defect causes the head to resemble the head of a pin or nail, which may be off-set or slanted. In these sperm, the head was unusually oblong in shape and much smaller than normal. Typically, pin-head sperm result when the centrioles from which the sperm tail develops are not correctly aligned opposite to the developing acrosome, so that the sperm head is lost and absorbed during epididymal transit [20].
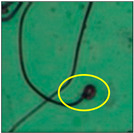 Knob-head	Amorphous head defects were also prevalent in the spermatozoa of the treated group, such as knob-shaped, umbrella-shaped or convex, and pointed-head sperm. All these spermatozoa had a small head region with no apical hook. These defects may have been due to disrupted spermatogenesis, which leads to the folding of the acrosome and the sperm head. This defect can make acrosome reactions impossible/abnormal, which eventually leads to hindering gamete interaction [21].
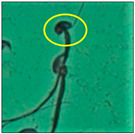 Umbrella-head	
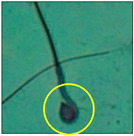 Pointed-head	
Various types of neck and mid-piece defects were observed in the mice of the experimental group.	
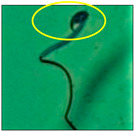 Bent-neck	Kinked or coiled necks werealso one of the major defects observed in the present study, with 163.8° bending. Varying degrees of neck bending could be seen, from slight to greater than 90°. Such a defect can occur due to missing centrioles, which form a guiding system for moving the chromosomes that are stored in the sperm neck. With this defect, the sperm cannot propel themselves, leading to the failure of gamete interaction [15].
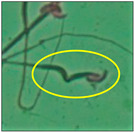 Spermatozoa with kink	Sharp bends or zigzags in the sperm mid-piece were noticed. This defect occurs when sperm contain abnormal mitochondrial sheaths, which cause the absence or lowering ofATP for sperm propulsion [22].
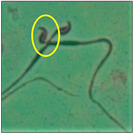 Abnormal neck attachment	A sperm with abnormal tail attachment to the head was observed. It was comparable to bent-neck sperm, but its neck and tail formed an angle of 90° (right angle). With a disoriented head and mid-piece, the penetration of the sperm is affected [22].
Various types of tail defects were observed in the mice of the experimental group.	
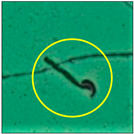 Brokentail	Tail stumps andshort tail lengths were also identified. Thishappens during late spermiogenesis. With such a structure, sperm lose their motility and fail to fertilize the ovum [23].
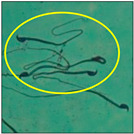 Distal benttail	Sharply bent tails, i.e., kinked tails, were observed.These present with bending or coiling near the tip of the tail. This defect causes abnormal sperm propulsion [24].
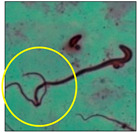 Schizoidtail	Sperm with two tails or incompletely dissociated tails that tether to a single tail were observed. Exposure to toxic chemicals and heavy metals can cause such defects, leading to abnormal movement and failure to successfully penetrate the egg [22].
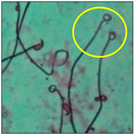 Hairpin loops	Coil-, loop-, or hairpin-like structures in the tails of sperm could be seen in the slides. In these sperm, the tail becomes bent, folding in on itself to form a loop (hairpin) structure. Spermatozoa with such a defect do not have normal movement [22].
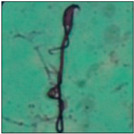 Highly coiled tail	Coiled tails were also visible in some places. Sometimes, such a severe abnormalityin the axial filament fibers causes the tail to become damaged, and the sperm cannot swim [25].
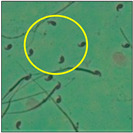 Sperm without tail	Tail-lesssperm, or acaudate sperm, were also observed in this study. Such spermatozoa are immotile [18]. The occurrence of this defect was quite high.
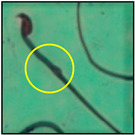 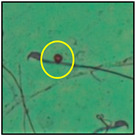 Light and heavy cytoplasmic droplets (CDs)	Light-type and heavy-type cytoplasmic droplets (CDs) were observed in the mid-pieces of the sperm. These can occur due to a lack of the Spem1 gene, obstructing proper cytoplasm removal and resulting in the bending of the tail and the wrapping of the midpiece, resulting in deficient sperm motility [26,27].
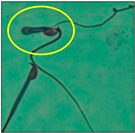 Fragmented/ degraded	Sperm with defective structures were also visible. These may have been caused by oxidative stress, infections, drug use, or exposure to environmental pollutants. These sperm had abnormally shaped acrosomes and damaged mid-pieces, which prevented acrosome reactions and eventually hindered gamete interaction [28].

**Table 3:** Morphometric measurements of spermatozoa.

Variable	Control Group	Experimental Group						
		Banana-Head	Hammer-Head	Large-Head	Headless	Pin-Head	Hookless	Umbrella-Head
**Head Length (µm)**	3.7 ± 0.19	6.8 ± 0.35 ***	2.7 ± 0.36 ***	4.1 ± 0.33	-	2.3 ± 0.09 ***	3.8 ± 0.09	2.02 ± 0.08 ***
**Head Width (µm)**	1.9 ± 0.37	1.5 ± 0.23	2.2 ± 0.34	2.3 ± 0.37	-	1.9 ± 0.19	1.7 ± 0.22	4.5 ± 0.18 ***
**Sperm Perimeter (µm)**	122.0 ± 4.04	116.8 ± 2.73	91.2 ± 2.63 ***	125.3 ± 1.79	114.5 ± 6.91	97.6 ± 3.61 ***	117.8 ± 1.61	90.7 ± 3.49 ***
**Sperm Area (µm^2^)**	47.1 ± 1.76	33.5 ± 3.81 ***	41.4 ± 2.76	49.5 ± 5.65	35.3 ± 6.51 **	20.57 ± 0.75 ***	32.1 ± 1.41 ***	46.2 ± 3.26

Values represent mean ± SD. While ** *p* < 0.01 has been considered very significant and *** *p* < 0.001 as highly significantly different. Comparisons were carried out using Student’s *t*-test, and significant differences among various groups were further analyzed using one-way ANOVA (Tukey’s) tests with GraphPad prism 8 software; ***: *p* ≤ 0.001.

## Data Availability

This manuscript has no associated data.
